# Plasma neurofilament light is a predictor of neurological outcome 12 h after cardiac arrest

**DOI:** 10.1186/s13054-023-04355-3

**Published:** 2023-02-24

**Authors:** Helena Levin, Anna Lybeck, Attila Frigyesi, Isabelle Arctaedius, Bergthóra Thorgeirsdóttir, Martin Annborn, Marion Moseby-Knappe, Niklas Nielsen, Tobias Cronberg, Nicholas J. Ashton, Henrik Zetterberg, Kaj Blennow, Hans Friberg, Niklas Mattsson-Carlgren

**Affiliations:** 1 Anesthesia & Intensive Care, Department of Clinical Sciences, Lund University, Lund, Sweden; 2 Department of Research & Education, Skane University Hospital, Lund, Sweden; 3 Anesthesia & Intensive Care, Department of Clinical Sciences, Skane University Hospital, Lund University, Lund, Sweden; 4 Anesthesia & Intensive Care, Department of Clinical Sciences, Skane University Hospital, Lund University, Malmö, Sweden; 5 Anesthesia & Intensive Care, Department of Clinical Sciences, Helsingborg Hospital, Lund University, Helsingborg, Sweden; 6 Neurology, Department of Clinical Sciences Lund, Skane University Hospital, Lund University, Lund, Sweden; 7 Institute of Psychiatry, Psychology and Neuroscience, King’s College London, London, UK; 8 NIHR Biomedical Research Centre for Mental Health and Biomedical Research Unit for Dementia at South London and Maudsley NHS Foundation, London, UK; 9 Centre for Age-Related Medicine, Stavanger University Hospital, Stavanger, Norway; 10 Department of Psychiatry and Neurochemistry, Institute of Neuroscience and Physiology, The Sahlgrenska Academy at the University of Gothenburg, Mölndal, Sweden; 11 Clinical Neurochemistry Laboratory, Sahlgrenska University Hospital, Mölndal, Sweden; 12 Department of Neurodegenerative Disease, UCL Institute of Neurology, Queen Square, London, UK; 13 UK Dementia Research Institute at UCL, London, UK; 14 Hong Kong Center for Neurodegenerative Diseases, Clear Water Bay, Hong Kong, China; 15 Clinical Memory Research Unit, Department of Clinical Sciences, Lund University, Malmö, Sweden; 16 Department of Neurology, Skane University Hospital, Lund, Sweden; 17 Wallenberg Center for Molecular Medicine, Lund University, Lund, Sweden

**Keywords:** Out-of-hospital cardiac arrest (OHCA), In-hospital cardiac arrest (IHCA), Prognostication, Biomarker, Neurofilament light (NfL)

## Abstract

**Background:**

Previous studies have reported high prognostic accuracy of circulating neurofilament light (NfL) at 24–72 h after out-of-hospital cardiac arrest (OHCA), but performance at earlier time points and after in-hospital cardiac arrest (IHCA) is less investigated. We aimed to assess plasma NfL during the first 48 h after OHCA and IHCA to predict long-term outcomes.

**Methods:**

Observational multicentre cohort study in adults admitted to intensive care after cardiac arrest. NfL was retrospectively analysed in plasma collected on admission to intensive care, 12 and 48 h after cardiac arrest. The outcome was assessed at two to six months using the Cerebral Performance Category (CPC) scale, where CPC 1–2 was considered a good outcome and CPC 3–5 a poor outcome. Predictive performance was measured with the area under the receiver operating characteristic curve (AUROC).

**Results:**

Of 428 patients, 328 (77%) suffered OHCA and 100 (23%) IHCA. Poor outcome was found in 68% of OHCA and 55% of IHCA patients. The overall prognostic performance of NfL was excellent at 12 and 48 h after OHCA, with AUROCs of 0.93 and 0.97, respectively. The predictive ability was lower after IHCA than OHCA at 12 and 48 h, with AUROCs of 0.81 and 0.86 (*p* ≤ 0.03). AUROCs on admission were 0.77 and 0.67 after OHCA and IHCA, respectively. At 12 and 48 h after OHCA, high NfL levels predicted poor outcome at 95% specificity with 70 and 89% sensitivity, while low NfL levels predicted good outcome at 95% sensitivity with 71 and 74% specificity and negative predictive values of 86 and 88%.

**Conclusions:**

The prognostic accuracy of NfL for predicting good and poor outcomes is excellent as early as 12 h after OHCA. NfL is less reliable for the prediction of outcome after IHCA.

**Supplementary Information:**

The online version contains supplementary material available at 10.1186/s13054-023-04355-3.

## Background

Accurate neuroprognostication is essential in patients who remain comatose after cardiac arrest to identify patients who may recover and avoid futile treatment in those who will never awake. Current guidelines recommend multimodal prognostication no earlier than 72 h post-arrest to allow time for recovery and clearance of sedative drugs [1]. Further investigation into markers with early prognostic value in comatose survivors after cardiac arrest may enable earlier multimodal neuroprognostication.

Neurofilament light chain protein (NfL) is a novel biomarker of neuroaxonal injury. Elevated circulating NfL at 24–72 h is highly predictive of poor neurological outcome after out-of-hospital cardiac arrest (OHCA) [2–4]. Additionally, low levels of NfL at 24–72 h have been reported to accurately identify patients with good outcome [3, 5]. The prognostic value is low early after ICU admission and the earliest time point when NfL can provide reliable prognostic information is unknown [3, 6]. Previous studies reporting the highest prognostic performance of NfL included only selected cohorts of OHCA [2, 3]. Further studies are needed to validate the predictive value of NfL in heterogeneous cohorts of post-cardiac arrest patients [7].

The current study aimed to investigate the prognostic performance of NfL to predict good and poor outcomes in the first two days after cardiac arrest in an unselected cohort of OHCA and in-hospital cardiac arrest (IHCA) patients. We hypothesised that the predictive performance would be acceptable at 12 h and improve at 48 h due to accumulating brain injury and biomarker kinetics. We also hypothesised that NfL would be a more accurate predictor of outcome after OHCA than IHCA due to differences in patient and clinical characteristics [8, 9].

## Methods

### Study design and setting

This was a retrospective, multicentre observational study of patients admitted after cardiac arrest to three Intensive Care Units (ICU) in southern Sweden from 2014 to 2018, with no pre-defined study size. The patients were consecutively included in the SWECRIT biobank, aiming to study biomarkers in critically ill patients (ClinicalTrials.gov no. NCT04974775, retrospectively registered July 2021). The study protocol was approved by the Regional Ethical Review Board in Lund, Sweden (registration no. 2014–47 and 2022-02681-01). Written informed consent was obtained from patients who regained mental capacity. The Standards for Reporting Diagnostic accuracy studies (STARD) guidelines were followed [10].

### Study population

All patients 18 years or older admitted to intensive care after cardiac arrest were eligible for inclusion. International guidelines on post-resuscitation care were followed, including multimodal neuroprognostication no earlier than 72 h after cardiac arrest for patients who remained unconscious [11]. Blood samples were collected on ICU admission, 12 and 48 h after cardiac arrest. Samples drawn within six hours of the specified time points were included for statistical analysis. Patients with admission samples only (0–6 h after cardiac arrest) were excluded from this study.

### Biochemical analyses 

All samples were centrifuged, aliquoted, and frozen to −80 °C before storage in the biobank at Region Skane, Sweden (BD-47, SC-1922). The measurements of NfL were performed at the Clinical Neurochemistry Laboratory at the University of Gothenburg in September 2021 by staff blinded to clinical outcomes. Plasma NfL levels were measured using a single-molecule array (Simoa) NfL immunoassay on an HD-X analyser according to instructions from the kit manufacturer (Quanterix, Billerica, MA). Serum neuron-specific enolase (NSE) was analysed by the local laboratory in Region Skane as part of management in clinical practice [12], using a Cobas instrument with electrochemiluminescent immunoassays (Roche Diagnostics, Rotkreuz, Switzerland).

### Data sources and outcome

Patient data were collected from the International Cardiac Arrest Registry (INTCAR), the patient administrative system for intensive care units (PASIVA), the Swedish population register, and medical records. Routine electroencephalogram (EEG) was performed when indicated at the treating physician’s discretion. Neurological outcome according to the Cerebral Performance Category (CPC) scale [13] was assessed two to six months post-arrest by personnel (physician or nurse) blinded to NfL levels. We considered CPC 1–2 as good outcome and CPC 3–5 as poor outcome.

### Statistical methods

Clinical characteristics are presented as medians and interquartile ranges (IQR) or as counts and percentages. Groups were compared using Mann–Whitney U test, Pearson’s Chi-squared test, or logistic regression. Diagnostic performance was assessed by the area under the receiver operating characteristic curve (AUROC), defined from logistic regression models with and without addition of alternative methods for prediction of outcome. A model with clinical data included age, time to ROSC, witnessed cardiac arrest, bystander CPR (OHCA), shockable rhythm, and administration of adrenaline. The DeLong method was used to compare AUROCs for paired data.

To account for missing data, a multiple imputations approach [14] using the package mice for R was used with the CART method (imputation by classification and regression trees) over *N* = 20 multiple imputations. Pooled AUROCs were calculated over the imputed datasets using Rubin’s Rules, as implemented in the psfmi package for R.

We assessed partial AUROC (pAUROC) to predict poor outcome at 95–100% specificity. We determined pAUROC to predict a good outcome at 80–95% sensitivity, aiming to be highly predictive and to identify most patients with a good outcome. At the set specificities (95 and 100%) and sensitivities (80 and 95%), we determined cut-offs, positive predictive values (PPV, prediction of poor outcome), and negative predictive values (NPV, prediction of good outcome). We used a bootstrap method implemented in the R package *pROC* to calculate 95% confidence intervals (CI) and compare paired pAUROCs.

We used logistic regression models separately for OHCA and IHCA to adjust for covariates identified with stepwise backward regression. The backward elimination was continued for as long as the Akaike information criterion (AIC) decreased (R package *MASS*). We also evaluated differences in the performance of NfL to predict outcome after OHCA and IHCA with an interaction model, testing the interaction between NfL and cardiac arrest setting (OHCA vs IHCA). We tested if the covariates identified in the previous step eliminated the interaction, first using all covariates and finally only the covariates with a significant effect in the interaction model.

To reduce the skewness of the NfL measurements, we used log10-transformed data. Significance was set at *p* < 0.05. All analyses were performed using R, version 4.1.2 [15].

## Results

### Study population

**Fig. 1:**
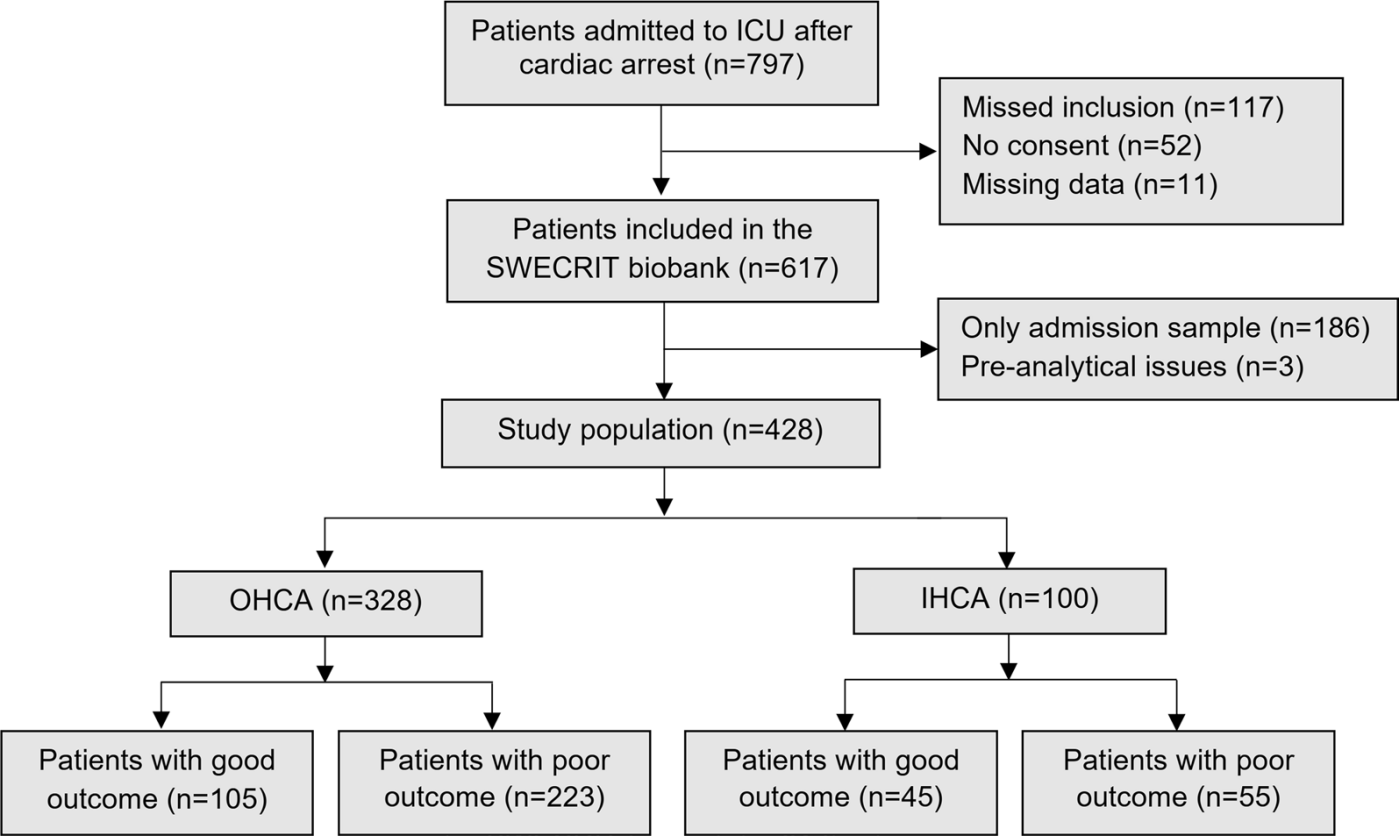
Study flowchart. Long-term outcomes, according to the Cerebral Performance Category (CPC) scale, were dichotomised into good (CPC 1–2) and poor (CPC 3–5) outcomes. *ICU* intensive care unit*, OHCA* out-of-hospital cardiac arrest, *IHCA* in-hospital cardiac arrest

Of 797 patients admitted after cardiac arrest, 617 patients were included in the SWECRIT biobank. After exclusions, 428 patients remained, and of those, 328 (77%) suffered OHCA and 100 (23%) IHCA (Fig. 1). A description of missed and excluded patients is presented in Additional file 1: Table S1. Patient characteristics and outcomes are presented in Table 1 and stratified by outcomes in Additional file 1: Table S2. IHCA compared to OHCA patients were more often females, had more often congestive heart failure, renal disease, diabetes, and cerebrovascular disease, less often a cardiac cause and shockable rhythm, shorter time to ROSC, and on ICU admission higher Glasgow Coma Scale Motor response (GCS-M) and lower lactate (*p* < 0.05). The mortality at 30 and 180 days were lower and withdrawal of life-sustaining therapy (WLST), WLST due to poor prognosis, and poor neurological outcome were less common after IHCA than OHCA (*p* < 0.01).

**Table 1:** Characteristics of OHCA and IHCA patients

	OHCA (*n* = 328)	IHCA (*n* = 100)
Age, years–median (IQR)	67 (59–75)	71 (59–77)
Sex, male–*n* (%)	247 (75)	63 (63)
Medical history		
Myocardial infarction–*n* (%)	50 (15)	19 (19)
Congestive heart failure–*n* (%)	52 (16)	26 (26)
Hypertension–*n* (%)	124 (38)	48 (48)
Liver disease–*n* (%)	6 (2)	4 (4)
Renal disease–*n* (%)	24 (7)	18 (18)
Diabetes–*n* (%)	66 (20)	37 (37)
Cerebrovascular disease–*n* (%)	25 (8)	15 (15)
Dementia/cognitive impairment–*n* (%)	13 (4)	6 (6)
Solid tumour–*n* (%)	31 (9.5)	17 (17)
Cardiac arrest characteristics		
Time to ROSC, min–median (IQR)	25 (15–40)	10 (6–20)
Witnessed cardiac arrest–*n* (%)	256 (78)	83 (83)
Bystander-performed CPR–*n* (%)	206 (63)	NA
Arrest with medical personnel present–*n* (%)	39 (12)	100 (100)
Shockable rhythm–*n* (%)	177 (54)^a^	21 (21)^a^
Adrenaline–*n* (%)	262 (80)	83 (83)
Cardiac cause–*n* (%)	246 (75)	38 (38)
Characteristics on ICU admission		
GCS-M–median (IQR)	1 (1–2)^e^	3 (1–5)^c^
Circulatory shock–*n* (%)	103 (31)	32 (32)
Lactate–median (IQR)	8.7 (5.3–11.6)	6.7 (4.7–9.9)
pH–median (IQR)	7.2 (7.0–7.3)	7.2 (7.0–7.3)^a^
Outcome		
ICU length of stay, days–median (IQR)	3.1 (1.9–5.0)	3.2 (1.4–4.9)
Hospital length of stay, days–median (IQR)	6.0 (3.0–12.0)^c^	9.5 (4.0–20.8)^b^
WLST–*n* (%)	181 (55)	34 (34)^a^
WLST due to poor neurological prognosis–*n* (%)	159 (48)^d^	26 (26)^b^
Mortality at 30 days–*n* (%)	214 (65)^a^	46 (46)^c^
Mortality at 180 days–*n* (%)	221 (67)^a^	53 (53)^c^
Time to follow-up, months–median (IQR)	4 (3–6)	6 (4–6)
CPC at follow-up–*n* (%)		
CPC 1	69 (21)	24 (24)
CPC 2	36 (11)	21 (21)
CPC 3	2 (1)	3 (3)
CPC 4	0 (0)	0 (0)
CPC 5	221 (67)	52 (52)

Proportions (%) are within the groups of OHCA and IHCA patients. Missing data: ^a^*n* = 1, ^b^*n* = 2, ^c^*n* = 3, ^d^*n* = 4, ^e^*n* = 5. *IQR* interquartile range, *ROSC* return of spontaneous circulation*,* *GCS-M* Glasgow Coma Scale Motor response, *ICU* intensive care unit, *CPC* Cerebral Performance Category*, WLST* withdrawal of life-sustaining therapy, *OHCA* out-of-hospital cardiac arrest, *IHCA* in-hospital cardiac arrest

**Fig. 2:**
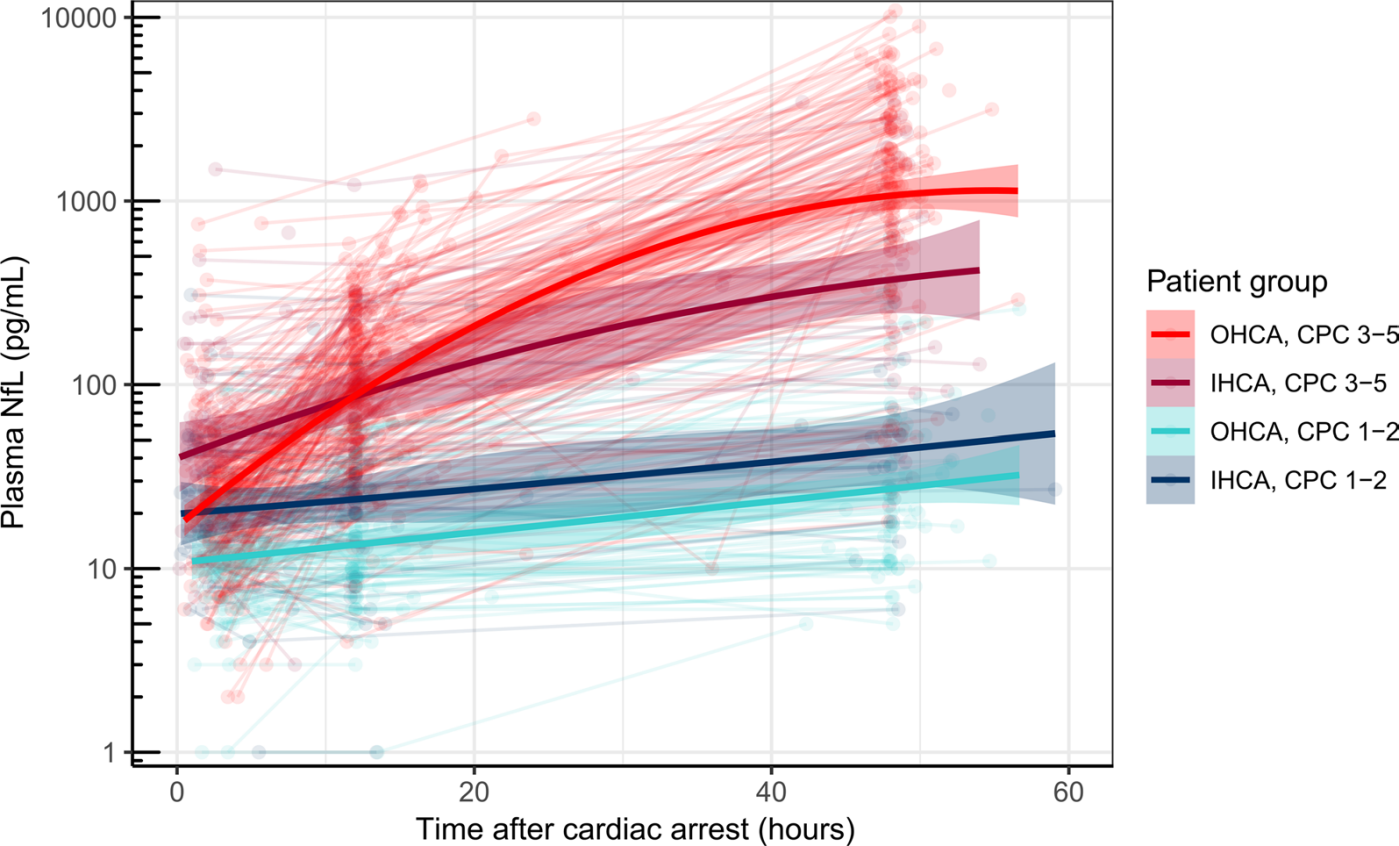
Neurofilament light (NfL) levels over time in OHCA and IHCA patients with good (CPC 1–2) and poor (CPC 3–5) outcomes. The bold lines show the trajectory trend with 95% CI. Each dot represents a sample, and each subtle line a patient. In addition to samples at the defined time points, admission (0–6 h), 12 ± 6 h, and 48 ± 6 h, samples collected outside these time limits (n = 60) were included in this illustration. *OHCA* out-of-hospital cardiac arrest, *IHCA* in-hospital cardiac arrest, *CPC* Cerebral Performance Category

NfL levels were available on admission in 289 OHCA and 83 IHCA patients, at 12 h in 300 OHCA and 87 IHCA patients, and at 48 h in 210 OHCA and 54 IHCA patients. The reasons for missed sampling and time to sampling are presented in Additional file 1: Table S3. In addition, NfL levels were available in 60 samples from 39 OHCA and 13 IHCA patients outside the defined time limits (these data were not used for statistical analyses, but included in Fig. 2).

### Plasma levels of NfL

NfL levels were higher in patients with poor compared to good outcome at all time points after both OHCA and IHCA, Additional file 1: Fig. S1. The difference remained significant after adjustment for covariates, (*p* < 0.02 on admission, *p* < 0.008 at 12 and 48 h) (see Additional file 1: Fig. S2). In patients with a good outcome, NfL median levels were lower after OHCA than IHCA on admission (10 vs. 22 pg/ml, *p* < 0.001) and at 12 h (11 vs. 25 pg/ml, *p* = 0.009) but not significantly different at 48 h (21 vs. 36 pg/ml). In patients with poor outcome, NfL levels were lower after OHCA than IHCA on admission (23 vs. 44 pg/ml, *p* = 0.001), similar at 12 h (96 vs. 100 pg/ml), and higher at 48 h (1425 vs. 541 pg/ml, *p* = 0.001). The trends of NfL levels over time in all OHCA and IHCA patients with good and poor outcomes are visualised in Fig. 2.

### Performance of NfL for prediction of outcomes

**Fig. 3:**
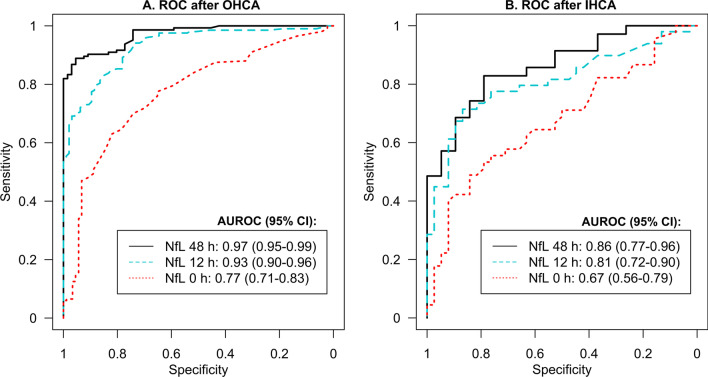
**A**-**B** Performance of neurofilament light (NfL) for prediction of neurologic outcomes. Receiver operating characteristic curves (ROC) and the area under the curve (AUROC) of NfL levels measured on admission (0 h), 12 h, and 48 h after OHCA and IHCA for prediction of good (CPC 1–2) versus poor (CPC 3–5) outcomes. *OHCA* out-of-hospital cardiac arrest, *IHCA* in-hospital cardiac arrest, *CPC* Cerebral Performance Category

The overall prognostic performance of NfL was excellent at 12 and 48 h after OHCA, with AUROCs of 0.93 and 0.97, respectively (*p* = 0.02) (Fig. 3A). After IHCA, the AUROCs at 12 and 48 h were 0.81 and 0.86, respectively (*p* = 0.44) (Fig. 3B). The predictive ability was higher after OHCA compared to IHCA at 12 h (*p* = 0.02) and 48 h (*p* = 0.03). The performance on admission did not differ significantly between OHCA and IHCA, with AUROCs of 0.77 and 0.67, respectively. In sensitivity analyses where the AUROCs for NfL to predict poor outcome were calculated over multiple imputed datasets in all included patients (*n* = 617, Fig. 1), the results were overall similar as in the main analyses (Additional file 1: Table S4).

### NfL combined with other data for prognostication

We also tested if NfL provided additional information to other prognostic modalities. The prognostic performance of a model with baseline clinical information increased when adding NfL at 12 h or 48 h after OHCA (from AUROCs 0.86–0.90 for clinical models to 0.96–0.98 for combined models) and after IHCA (from AUROCs 0.72–0.73 to 0.85–0.96) (*p* < 0.01), but not when adding NfL on admission (Additional file 1: Table S5).

In a subgroup of OHCA patients with EEG data (not analysed in IHCA due to limited data), the prognostic performance of routine EEG (median [IQR] 75 [59-98] h after cardiac arrest) was improved by adding NfL at 12 h (AUROC 0.77 to 0.90, *p* < 0.001) or 48 h (AUROC 0.74 to 0.97, *p* < 0.001), but not by NfL on admission (Additional file 1: Table S6).

In a subgroup of OHCA and IHCA patients with available NSE data (sampled at 24 h and 48 h), NfL at 12 h was superior to NSE at 24 h, while NfL at 48 h was superior to NSE at 48 h (*p* < 0.001 after OHCA, *p* < 0.05 after IHCA), (Additional file 1: Fig. S3).

### Exploring the different prognostic abilities of NfL in OHCA and IHCA

Due to the differences in results for OHCA and IHCA described above, we evaluated an interaction model where NfL and cardiac arrest setting (OHCA or IHCA) showed a significant interaction to predict poor outcome, when using NfL at 12 h (*p* = 0.003) but not NfL on admission or at 48 h. The interaction at 12 h was eliminated (*p* = 0.17) by adding the significant covariates age, time to ROSC, cardiac cause, shockable rhythm, and administration of adrenaline to the model (Additional file 1: Table S7).

### Plasma NfL for prediction of poor outcome

The performance of NfL to predict the outcome at high specificity (95–100%) assessed with pAUROC was higher after OHCA compared to IHCA at 12 and 48 h (*p* < 0.04) and similar on admission (Table 2). In OHCA patients, the pAUROC was lower at 12 h than at 48 h (*p* < 0.001). At 95% specificity, the sensitivity was 70% at 12 h and 89% at 48 h after OHCA.

**Table 2:** Accuracy of NfL at 95 and 100% specificity for prediction of poor outcome

	Time point	N	95% specificity				100% specificity				
			Sensitivity %	Cut-off, pg/mL	NPV %	PPV %	Sensitivity %	Cut-off, pg/mL	NPV %	PPV %	pAUROC 95–100%
OHCA	0 h	289	15 (5–53)	59	33	87	6 (3–14)	118	32	100	0.53
	12 h	300	70 (56–78)	61	60	97	53 (47–71)	90	50	100	0.81
	48 h	210	89 (81–94)	220	80	97	82 (76–90)	336	72	100	0.92
IHCA	0 h	83	22 (0–51)	114	49	83	4 (0–42)	393	47	100	0.54
	12 h	87	45 (20–73)	133	57	92	29 (18–63)	207	52	100	0.67
	48 h	54	57 (34–77)	323	50	95	49 (34–77)	640	51	100	0.74

Sensitivities with 95% CI, cut-offs, negative predictive values (NPV), and positive predictive values (PPV) at pre-defined high specificities (95% and 100%) for neurofilament light (NfL) on admission (0 h), 12 and 48 h after OHCA and IHCA. The pAUROC presents the performance of NfL in the 95–100% specificity range. A 95% specificity indicates that 5% of the patients with good outcome had NfL levels above the cut-off. *OHCA* out-of-hospital cardiac arrest, *IHCA* in-hospital cardiac arrest, *pAUROC* partial area under the receiver operating curve

### Plasma NfL for prediction of good outcome

The performance of low NfL levels to predict the outcome at high sensitivity (80–95%) was higher after OHCA compared to IHCA at 12 h and 48 h (*p* < 0.03) and similar on admission (Table 3). In OHCA patients, there was no significant difference in performance between NfL at 12 h and 48 h. At 95% sensitivity in OHCA at 12 h and 48 h, 71% and 74% of the patients with good outcome (specificity) had NfL levels below the cut-off, corresponding to 86% and 88% correct prediction of good outcome (NPV).

**Table 3:** Accuracy of NfL at 95 and 80% sensitivity for prediction of good outcome

	Time point	N	95% sensitivity				80% sensitivity				
			Specificity %	Cut-off, pg/mL	NPV%	PPV%	Specificity %	Cut-off, pg/mL	NPV%	PPV%	pAUROC 80–95%
OHCA	0 h	289	20 (7–31)	7	62	73	60 (45–72)	12	56	82	0.66
	12 h	300	71 (59–81)	23	86	87	88 (77–95)	45	67	93	0.88
	48 h	210	74 (64–88)	53	88	89	100 (94–100)	388	69	100	0.95
IHCA	0 h	83	16 (3–26)	9	75	57	37 (11–55)	16	64	61	0.55
	12 h	87	13 (0–47)	9	63	58	55 (29–92)	27	68	70	0.64
	48 h	54	37 (11–68)	30	78	73	79 (42–95)	91	68	88	0.75

Specificities with 95% CI, cut-offs, negative predictive values (NPV), and positive predictive values (PPV) at pre-defined high sensitivities (95% and 80%) for neurofilament light (NfL) on admission (0 h), 12 and 48 h after OHCA and IHCA. The pAUROC presents performance of NfL in the 80–95% sensitivity range. A 95% sensitivity indicates that 5% of the patients with poor outcome had NfL levels below the cut-off. *OHCA* out-of-hospital cardiac arrest, *IHCA* in-hospital cardiac arrest, *pAUROC* partial area under the receiver operating curve

## Discussion

This multicentre cohort study investigated the ability of plasma NfL to predict long-term neurological outcome in a heterogeneous population admitted to intensive care after cardiac arrest. NfL at 12 and 48 h after OHCA reliably predicted good and poor neurological outcomes. The predictive ability after IHCA compared to OHCA was lower at 12 and 48 h.

The prognostic performance of NfL at 12 h after OHCA in this study was similar to the accuracy of NfL at 24 and 48 h in two previous studies [2, 4]. One study included an unselected OHCA cohort [4], and the other investigated a selected group of OHCA patients of a presumed cardiac cause [2, 16]. The performance was slightly higher in the COMACARE trial [3, 17], which included a more selected patient group with initial shockable rhythm. The accuracy of any test will depend on the population studied, and the predictive ability will likely improve in highly selected populations of OHCA. Despite the heterogeneous OHCA cohort in this study, the ability of NfL to predict the outcome at 12 and 48 h was excellent, and superior to NSE. This novel finding supports the generalisability of NfL as a useful prognostic tool across the entire spectrum of OHCA patients in the ICU.

In the present study, the prediction of poor outcome at high specificity was better at 48 h compared to 12 h after OHCA. The improved prediction with time likely reflects evolving post-anoxic brain injury but may also be explained by patients not included at 48 h due to prior haemodynamic collapse and circulatory deaths. Although lower than at 48 h, the predictive ability of NfL at 12 h shown here may still be of clinical value.

Low levels of NfL have been shown to predict a good neurological outcome at 24 h after OHCA [3, 5]. In this study, NfL, as early as 12 h after OHCA, had a high ability to predict good outcome. Early prediction of neurological recovery may aid in decision-making, e.g. regarding invasive procedures, and could help avoid inappropriate limitations in care and provide relevant information for relatives. Another potential clinical use of a low 12 h NfL value would be to tailor neuroprotective strategies, e.g. by attempting an early wake up in patients with low NfL. Our results suggest that the prognostic value of EEG is improved by NfL already at 12 h, supporting the prognostic performance of NfL when measured at least 12 h after cardiac arrest. Future studies could explore whether early prediction of outcome may be further strengthened by combining NfL levels at 12 or 24 h with an early (< 24 h) return of a continuous background pattern on EEG [18, 19].

In this study, the predictive performance of NfL on admission to intensive care was low, which is in line with previous studies [3, 6, 20]. In patients with poor outcome, NfL levels increase substantially as post-hypoxic brain injury develops and may reach a steady state between 48 and 72 h [2, 3, 21]. NfL is also known to increase with age, even under normal circumstances [22], and elevated NfL levels can be caused by neurological comorbidity and traumatic brain injury [23–27]. NfL increase caused by other conditions is typically low compared to NfL levels released due to hypoxic brain injury [28–30], but in very early prognostication and when low NfL levels are used to predict a good outcome, other causes of NfL elevation may be important to consider.

To our knowledge, NfL has previously not been analysed in an IHCA cohort. The prognostic accuracy of NfL was lower in IHCA compared to OHCA at both 12 and 48 h. However, a significant interaction between the location of arrest and outcome prediction by NfL was only found at 12 h. These results suggest an overall trend for lower predictive ability after IHCA, particularly early after cardiac arrest. IHCA and OHCA patients differ in key parameters that are relevant for prognostication. For example, the time to ROSC was shorter and WLST due to poor prognosis less common after IHCA than OHCA, suggesting a less severe brain injury in IHCA. This is also reflected by the higher level of consciousness on ICU admission after IHCA. When focusing on patients with good outcome, we found that NfL was generally higher in IHCA than OHCA, which may be explained by their higher age and more comorbidities in this cohort [8, 9, 31]. NfL, as a marker of neuroaxonal injury, is not likely to identify patients with poor outcomes due to haemodynamic collapse or other causes of non-neurological death, which may contribute to the lower predictive ability of NfL in IHCA patients. When adjusting our analyses for age, time to ROSC, cardiac cause, shockable rhythm and administration of adrenaline, the difference between IHCA and OHCA in predictive ability of NfL was no longer significant, suggesting that patient and cardiac arrest characteristics may explain the lower predictive value of NfL after IHCA.

## Strengths and limitations

Strengths of the present study are the heterogeneous cohort, allowing for more generalisable results, the multicentre design, the large sample size of OHCA patients, and the batch analysis of NfL samples. A limitation of this and other NfL studies is that there is no certified standard for analysis, and both analytical and preanalytical factors may vary, complicating comparisons of NfL levels reported by different studies. Before NfL can be implemented in clinical practice, future studies must establish a standard for analysis and normal values for NfL. This study is also limited by its retrospective design. The data quality depended on the documentation in the patient's medical notes, data on the presumed cause of death was unavailable, and the time to follow-up varied. The sample size of IHCA was small, particularly at 48 h.

Another limitation is that a lack of withdrawal of consent after regaining consciousness in some patients will lead to an underestimation of the proportion of survivors. Differences between included and excluded patients suggest that missing patients were due to early deaths and transfers from the ICU. Prognostication is not performed in clinical practice in patients who die or wake up early, but we cannot exclude that missed inclusions have affected the generalisability of the study.

## Conclusion

This study suggests that NfL accurately predicts good and poor neurological outcomes as early as 12 h after cardiac arrest in a heterogeneous group of OHCA patients. The predictive ability of NfL is less reliable after IHCA and needs further investigation.

## Supplementary Information

**Additional file 1**. Supplementary tables and figures.

## Data Availability

The study protocol and the datasets analysed during the current study are available from the corresponding author on reasonable request.

## Abbreviations

AIC: Akaike information criterion
AUROC: Area under the receiver operating curve
CI: Confidence interval
COPD: Chronic obstructive pulmonary disease
CPC: Cerebral Performance Category
CPR: Cardiopulmonary resuscitation
EEG: Electroencephalogram
ICU: Intensive care unit
GCS-M: Glasgow Coma Scale Motor response
IHCA: In-hospital cardiac arrest
INTCAR: International cardiac arrest registry
IQR: Interquartile range
NfL: Neurofilament light chain
NPV: Negative predictive values
NSE: Neuron-specific enolase
OHCA: Out-of-hospital cardiac arrest
OR: Odds ratio
PASIVA: Patient administrative system for intensive care units
pAUROC: Partial area under the receiver operating curve
PPV: Positive predictive values
ROSC: Return of spontaneous circulation
Simoa: Single-molecule array
STARD: Standards for reporting diagnostic accuracy studies
WLST: Withdrawal of life-sustaining therapy
