# Supplementary material for: Plasma neurofilament light is a predictor of neurological outcome 12 h after cardiac arrest
Source: Crit Care. 2023 Feb 24;27:74. doi: 10.1186/s13054-023-04355-3 (PMC9960417; doi:10.1186/s13054-023-04355-3)
Supplement: Supplementary file 1 — Additional file 1. Supplementary tables and figures. [file 13054_2023_4355_MOESM1_ESM.docx]

Supplementary Material

**Plasma neurofilament light is a predictor of neurological outcome 12 h after cardiac arrest**

**Authors**

Helena Levin^1,2*^, Anna Lybeck^3^, Attila Frigyesi^3^, Isabelle Arctaedius^3^, Bergthóra Thorgeirsdóttir^4^, Martin Annborn^5^, Marion Moseby-Knappe^6^, Niklas Nielsen^5^, Tobias Cronberg^6^, Nicholas J. Ashton^7-10^, Henrik Zetterberg^10-14^, Kaj Blennow^10,11^, Hans Friberg^4^, Niklas Mattsson-Carlgren^15-17^

**Affiliations**

^1^ Anesthesia & Intensive Care, Department of Clinical Sciences, Lund University, Lund, Sweden.

^2^ Department of Research & Education, Skane University Hospital, Lund, Sweden.

^3^ Anesthesia & Intensive Care, Department of Clinical Sciences, Lund University, Skane University Hospital, Lund, Sweden.

^4^ Anesthesia & Intensive Care, Department of Clinical Sciences, Lund University, Skane University Hospital, Malmö, Sweden.

^5^ Anesthesia & Intensive Care, Department of Clinical Sciences, Lund University, Helsingborg Hospital, Helsingborg, Sweden.

^6^ Neurology, Department of Clinical Sciences Lund, Lund University, Skane University Hospital, Lund, Sweden.

^7^ Institute of Psychiatry, Psychology and Neuroscience, King’s College London, London, UK.

^8^ NIHR Biomedical Research Centre for Mental Health and Biomedical Research Unit for Dementia at South London and Maudsley NHS Foundation, London, UK.

^9^ Centre for Age-Related Medicine, Stavanger University Hospital, Stavanger, Norway.

^10^ Department of Psychiatry and Neurochemistry, Institute of Neuroscience and Physiology, the Sahlgrenska Academy at the University of Gothenburg, Mölndal, Sweden.

^11^ Clinical Neurochemistry Laboratory, Sahlgrenska University Hospital, Mölndal, Sweden.

^12^ Department of Neurodegenerative Disease, UCL Institute of Neurology, Queen Square, London, UK.

^13^ UK Dementia Research Institute at UCL, London, UK.

^14^ Hong Kong Center for Neurodegenerative Diseases, Clear Water Bay, Hong Kong, China.

^15^ Clinical Memory Research Unit, Department of Clinical Sciences, Lund University, Malmö, Sweden.

^16^ Department of Neurology, Skane University Hospital, Lund, Sweden.

^17^ Wallenberg Center for Molecular Medicine, Lund University, Lund, Sweden.

^*^ Correspondence: helena.levin@med.lu.se

**Table of Contents**

Table S1: Characteristics of study population compared to not included patients **3**

Table S2: Patient characteristics in OHCA and IHCA stratified by outcome**4**

Table S3: Available NfL results and reasons for missing samples**6**

Table S4: Sensitivity analysis of NfL for prediction of outcome**7**

Table S5: Prognostic performance of clinical data with and without NfL**8**

Table S6: Prognostic performance of EEG with and without NfL**9**

Table S7: Interaction model for the effect of NfL to prognosticate outcome**10**

Fig. S1: Boxplots of plasma NfL levels in patients with good vs poor outcome**11**

Fig. S2: Covariates for prediction of outcome after OHCA and IHCA**12**

Fig. S3: Prognostic performance of NfL compared to NSE**13**

**Table S1 Characteristics of study population compared to not included patients**

|  | **Study population (n=428)** | **Not included patients (n=369)** | ***p*-value** |
| --- | --- | --- | --- |
| Age, years - median (IQR) | 69 (59-76) | 71 (62-79) | 0.009 |
| Sex, male - n (%) | 310 (72) | 252 (68) | 0.230 |
| Alive at 30 days - n (%) | 168 (39) | 157 (45)^a^ | <0.001 |
| Alive at 180 days - n (%) | 154 (36) | 141 (40)^a^ | <0.001 |
| Survival days - median (IQR)^b^ | 4 (2-8) | 1 (0-11.5) | <0.001 |
| Length of ICU stay, days - median (IQR) | 3.1 (1.8-5.0) | 0.8 (0.3-3.0) | <0.001 |

A total of 797 patients were admitted to the study sites after cardiac arrest according to the patient administrative system for intensive care units (PASIVA) with the presented patient characteristics. Of all patients, 369 were excluded, or inclusion was missed. A flowchart with detailed information is shown in Figure 1. The *p*-values indicate the difference between the included study population vs the patients who were not included. *IQR* interquartile range*, ICU* intensive care unit

^a^ Missing survival data for 16 patients, % calculated on available data n= 353

^b^ Survival days for patients who died within six months

**Table S2 Patient characteristics in OHCA and IHCA stratified by outcomes**

|  | **OHCA, CPC 1-2** | **OHCA, CPC 3-5** | **IHCA, CPC 1-2** | **IHCA, CPC 3-5** | **OHCA vs IHCA**  **p-value** |
| --- | --- | --- | --- | --- | --- |
| Number of patients - n | 105 | 223 | 45 | 55 |  |
| Age, years - median (IQR) | 63.9 (53.3-72.8) | 69.4 (61.4-76.9) | 69.7 (57.3-76.1) | 73.4 (62.5-77.3) | 0.09 |
| Sex, male - n (%) | 89 (84.8) | 158 (70.9) | 26 (57.8) | 37 (67.3) | 0.02 |
| **Medical history** |  |  |  |  |  |
| Myocardial infarction - n (%) | 21 (20.0) | 29 (13.0) | 8 (17.8) | 11 (20.0) | 0.46 |
| Congestive heart failure - n (%) | 17 (16.2) | 35 (15.7) | 12 (26.7) | 14 (25.5) | 0.03 |
| Hypertension - n (%) | 35 (33.3) | 89 (39.9) | 21 (46.7) | 27 (49.1) | 0.09 |
| Liver disease - n (%) | 2 (1.9) | 4 (1.8) | 3 (6.7) | 1 (1.8) | 0.38 |
| Renal disease - n (%) | 2 (1.9) | 22 (9.9) | 9 (20.0) | 9 (16.4) | 0.003 |
| Diabetes - n (%) | 13 (12.4) | 53 (23.8) | 18 (40.0) | 19 (34.5) | 0.001 |
| Cerebrovascular disease - n (%) | 6 (5.7) | 19 (8.5) | 6 (13.3) | 9 (16.4) | 0.04 |
| Dementia/cognitive impairment - n (%) | 1 (1.0) | 12 (5.4) | 2 (4.4) | 4 (7.3) | 0.56 |
| Solid tumour - n (%) | 10 (9.5) | 21 (9.4) | 7 (15.6) | 10 (18.2) | 0.06 |
| **Cardiac arrest characteristics** |  |  |  |  |  |
| Time to ROSC, min – median (IQR) | 15.0 (10.0-25.0) | 30.0 (20.0-45.0) | 10.0 (5.0-20.0) | 12.0 (9.5-20.0) | <0.001 |
| Witnessed cardiac arrest – n (%) | 88 (83.8) | 168 (75.3) | 41 (91.1) | 42 (76.4) | 0.35 |
| Bystander-performed CPR | 78 (74.3) | 128 (57.4) | 0 (0.0) | 0 (0.0) | <0.001 |
| Arrest with medical personnel | 11 (10.5) | 28 (12.6) | 45 (100.0) | 55 (100.0) | <0.001 |
| Shockable rhythm - n (%) | 91 (87.5) | 86 (38.6) | 14 (31.1) | 7 (13.0) | <0.001 |
| Adrenaline - n (%) | 50 (47.6) | 212 (95.1) | 32 (71.1) | 51 (92.7) | 0.49 |
| Cardiac cause - n (%) | 98 (93.3) | 148 (66.4) | 23 (51.1) | 15 (27.3) | <0.001 |
| **Characteristics on ICU admission** |  |  |  |  |  |
| GCS-M - median (IQR) | 3.0 (1.0-4.5) | 1.0 (1.0-1.0) | 5.0 (2.0-6.0) | 1.0 (1.0-4.0) | <0.001 |
| Circulatory shock - n (%) | 18 (17.1) | 85 (38.1) | 14 (31.1) | 18 (32.7) | 1.00 |
| Lactate, mmol/L - median (IQR) | 5.9 (2.9-9.0) | 9.3 (6.6-12.1) | 5.9 (3.7-9.6) | 7.1 (5.2-10.1) | 0.005 |
| pH - median (IQR) | 7.3 (7.2-7.3) | 7.1 (6.9-7.2) | 7.2 (7.1-7.3) | 7.2 (7.0-7.3) | 0.24 |

**Table S2 (continued)**

|  | **OHCA, CPC 1-2** | **OHCA, CPC 3-5** | **IHCA, CPC 1-2** | **IHCA, CPC 3-5** | **OHCA vs IHCA**  **p-value** |
| --- | --- | --- | --- | --- | --- |
| **Outcome** |  |  |  |  |  |
| ICU length of stay, days - median (IQR) | 2.1 (1.7-3.7) | 3.6 (2.2-5.3) | 2.0 (1.0-4.0) | 4.0 (2.1-5.3) | 0.67 |
| Hospital length of stay, days - median (IQR) | 14.0 (10.0-25.0) | 4.00 (2.0-7.0) | 13.5 (10.5-31.3) | 5.0 (3.0-10.0) | 0.001 |
| WLST - n (%) | 0 (0.0) | 181 (81.2) | 0 (0.0) | 34 (61.8) | <0.001 |
| Mortality at 30 days - n (%) | 0 (0.0) | 214 (96.0) | 0 (0.0) | 46 (83.6) | 0.001 |
| Mortality at 180 days - n (%) | 0 (0.0) | 221 (99.1) | 2 (4.4) | 51 (92.7) | 0.01 |
| CPC at follow-up - n (%) |  |  |  |  | 0.005 |
| CPC 1 | 69 (65.7) | 0 (0.0) | 24 (53.3) | 0 (0.0) |  |
| CPC 2 | 36 (34.3) | 0 (0.0) | 21 (46.7) | 0 (0.0) |  |
| CPC 3 | 0 (0.0) | 2 (0.9) | 0 (0.0) | 3 (5.5) |  |
| CPC 4 | 0 (0.0) | 0 (0.0) | 0 (0.0) | 0 (0.0) |  |
| CPC 5 | 0 (0.0) | 221 (99.1) | 0 (0.0) | 52 (94.5) |  |

Long-term neurological outcome was dichotomised into good (CPC 1-2) and poor (CPC 3-5). Missing data in; shockable rhythm: OHCA n=1, IHCA n=1; GCS-M: OHCA n=5, IHCA n=3; pH: IHCA n=1; Hospital length of stay: OHCA n=3, IHCA n=2; WLST: IHCA n=1; Mortality at 30 and 180 days: OHCA n=1, IHCA n=3. *IQR* interquartile range, *ROSC* return of spontaneous circulation*, GCS-M* Glasgow Coma Scale Motor response, *ICU* intensive care unit, *CPC* Cerebral Performance Category*,*

**Table S3. Available NfL results and reasons for missing samples**

|  | **Time point** | **NfL results, n (%)** | **Time to sample, median (IQR), hours** | **Dead/ moribund, n (%)** | **Patient**  **not in ICU,**  **n (%)** | **Sampling out of time limit, n (%)** | **Other,**  **n (%)** |
| --- | --- | --- | --- | --- | --- | --- | --- |
| **OHCA** (n=328) | 0 h | 289 (88) | 2.8 (2.0-3.7) | 0 (0) | 3 (1) | 7 (2) | 29 (9) |
|  | 12 h | 300 (91) | 12.1 (11.9-12.9) | 0 (0) | 6 (2) | 8 (2) | 14 (4) |
|  | 48 h | 210 (64) | 48.1 (48.0-48.8) | 54 (16) | 37 (11) | 6 (2) | 21 (6) |
| **IHCA**  (n=100) | 0 h | 83 (83) | 1.6 (0.9-2.2) | 0 (0) | 2 (2) | 5 (5) | 10 (10) |
|  | 12 h | 87 (87) | 12.0 (11.8-12.7) | 0 (0) | 1 (1) | 2 (2) | 10 (10) |
|  | 48 h | 54 (54) | 48.2 (47.9-48.7) | 8 (8) | 27 (27) | 4 (4) | 7 (7) |

Available NfL results and the median time to sample after cardiac arrest. For missing samples the main reason was recorded. Missed sample due to sampling outside the time limit was not registered when a sample was available within defined time limits (admission [0-6 h after cardiac arrest], 12 ± 6 h, and 48 ± 6 h) or late due to the patient not being in the ICU. *NfL* neurofilament light, *OHCA* out-of-hospital cardiac arrest, *IHCA* in-hospital cardiac arrest, *IQR* interquartile range, *ICU* intensive care unit

**Table S4 Sensitivity analysis of NfL for prediction of outcome**

|  | **Time point** | **With imputations** | | **With original data** | |
| --- | --- | --- | --- | --- | --- |
|  |  | **N** | **AUROC (95% CI)** | **N** | **AUROC (95% CI)** |
| OHCA | 0 h | 435 | 0.75 (0.68-0.80) | 289 | 0.77 (0.71-0.83) |
|  | 12 h | 402 | 0.92 (0.89-0.95) | 300 | 0.93 (0.90-0.96) |
|  | 48 h | 340 | 0.96 (0.94-0.98) | 210 | 0.97 (0.95-0.99) |
| IHCA | 0 h | 182 | 0.68 (0.57-0.77) | 83 | 0.67 (0.56-0.79) |
|  | 12 h | 162 | 0.85 (0.77-0.91) | 87 | 0.81 (0.72-0.90) |
|  | 48 h | 146 | 0.92 (0.85-0.96) | 54 | 0.86 (0.77-0.96) |

Performance of neurofilament light (NfL) for prediction of neurologic outcomes with original data (as presented in Fig. 3) and with data after multiple imputations. 617 patients (435 OHCA and 182 IHCA) were included in the Swecrit biobank. Note that data were not imputed for patients who were dead at the time of sampling. The area under the receiver operating characteristic curve (AUROC) of NfL levels measured on admission (0 h), 12 h, and 48 h after OHCA and IHCA for prediction of good (CPC 1-2) versus poor (CPC 3-5) outcomes. *OHCA* out-of-hospital cardiac arrest, *IHCA* in-hospital cardiac arrest, *CPC* Cerebral Performance Category

**Table S5. Prognostic performance of clinical data with and without NfL**

|  | **Time point** | **N** | **AUROC (95% CI)**  **Clinical** | **AUROC (95% CI)**  **NfL** | **AUROC (95% CI)**  **Combined** | **p-value**  **Clinical vs Combined** | **p-value**  **NfL vs Combined** |
| --- | --- | --- | --- | --- | --- | --- | --- |
| OHCA | 0 h | 289 | 0.89 (0.85-0.93) | 0.77 (0.71-0.83) | 0.90 (0.87-0.94) | 0.09 | <0.001 |
|  | 12 h | 299 | 0.90 (0.86-0.94) | 0.93 (0.90-0.96) | 0.96 (0.94-0.98) | <0.001 | 0.002 |
|  | 48 h | 210 | 0.86 (0.81-0.92) | 0.97 (0.95-0.99) | 0.98 (0.97-0.99) | <0.001 | <0.05 |
| IHCA | 0 h | 82 | 0.74 (0.64-0.85) | 0.67 (0.55-0.79) | 0.79 (0.70-0.89) | 0.10 | 0.03 |
|  | 12 h | 87 | 0.73 (0.62-0.83) | 0.81 (0.72-0.90) | 0.85 (0.77-0.93) | 0.008 | 0.14 |
|  | 48 h | 53 | 0.72 (0.57-0.87) | 0.87 (0.77-0.96) | 0.96 (0.91-1.00) | <0.001 | 0.04 |

Predictive performance for clinical data with and without neurofilament light (NfL) on admission (0 h), at 12 h, and at 48 h after cardiac arrest. The clinical data in the model is age, time to ROSC, witnessed cardiac arrest, bystander CPR in OHCA, administration of adrenaline, and shockable rhythm. At each time point, only patients with both available clinical data and NfL were included. Since different datasets were used, the AUROCs for clinical data differ slightly between the time points. *OHCA* out-of-hospital cardiac arrest, *IHCA* in-hospital cardiac arrest, *AUROC* area under the receiver operating characteristic curve

**Table S6. Prognostic performance of EEG with and without NfL**

| **Time point**  **NfL** | **Time to EEG,**  **hours after OHCA**  **median (IQR)** | **N (CPC3-5)** | **AUROC (95% CI)**  **NfL** | **AUROC (95% CI) EEG** | **AUROC (95% CI)**  **NfL+EEG** | **p-value**  **EEG vs EEG+NfL** | **p-value**  **NfL vs EEG+NfL** |
| --- | --- | --- | --- | --- | --- | --- | --- |
| 0 h | 76 (59-99) | 143 (91) | 0.62 (0.46-0.78) | 0.76 (0.71-0.80) | 0.80 (0.71-0.89) | 0.32 | <0.001 |
| 12 h | 75 (61-94) | 144 (91) | 0.85 (0.79-0.92) | 0.77 (0.73-0.81) | 0.90 (0.84-0.95) | <0.001 | 0.009 |
| 48 h | 76 (63-100) | 125 (92) | 0.95 (0.92-0.99) | 0.74 (0.70-0.79) | 0.97 (0.94-1) | <0.001 | 0.11 |

Predictive performance of electroencephalogram (EEG) with and without neurofilament light (NfL) on admission (0 h), at 12 h, and at 48 h after OHCA. At each time point, only patients with both available EEG data and NfL were included. Since different datasets were used, the AUROCs for EEG data differ slightly between the time points. The cohort of in-hospital patients was not analysed due to a low number of performed EEGs in patients with NfL samples and only one patient with a good outcome (CPC1-2). *OHCA* out-of-hospital cardiac arrest, *IQR* interquartile range, *CPC* Cerebral Performance Category, *AUROC* area under the receiver operating characteristic curve

**Table S7 Interaction model for the effect of NfL at 12 h to prognosticate outcome**

| **Variable** | **OR (95% CI)** | ***p*-value** |
| --- | --- | --- |
| NfL 12 h (pg/mL) | 17.39 (4.54-88.70) | <0.001* |
| Location of arrest, OHCA | 0.56 (0.03-13.53) | 0.71 |
| Age (years) | 1,04 (1.01-1.07) | 0.02 * |
| Sex, male | 0.98 (0.43-2.20) | 0.96 |
| Previously healthy | 0.77 (0.31-1.89) | 0.56 |
| Renal disease | 0.54 (0.17-1.84) | 0.32 |
| Solid tumour | 1.16 (0.42-3.25) | 0.78 |
| Time to ROSC (min) | 1.05 (1.02-1.08) | 0.002* |
| Time to advanced support (min) | 0.99 (0.92-1.06) | 0.73 |
| Shockable rhythm | 0.40 (0.16-0.95) | 0.04 * |
| Administration of adrenaline | 3.35 (1.36-8.71) | 0.01 * |
| Circulatory shock | 2.19 (1.00-4.99) | 0.05 |
| Cardiac cause | 0.32 (0.12-0.79) | 0.02* |
| NfL 12 h (pg/mL) * location of arrest | 3.01 (0.43- 19.0) | 0.25 |

The interaction model for NfL and location of arrest (OHCA or IHCA) includes covariates identified by stepwise regression to prognosticate outcome. NfL and arrest location had significant interaction in predicting outcome with NfL at 12 h (*p*=0.003). The interaction effect was eliminated first using a model including all identified covariates (*p*=0.25) and finally with a model including only the significant (*p*<0.05*) covariates presented in this table (*p*=0.17). *OR* odds ratio, *NfL* neurofilament light, *OHCA* out-of-hospital cardiac arrest, *IHCA* in-hospital cardiac arrest


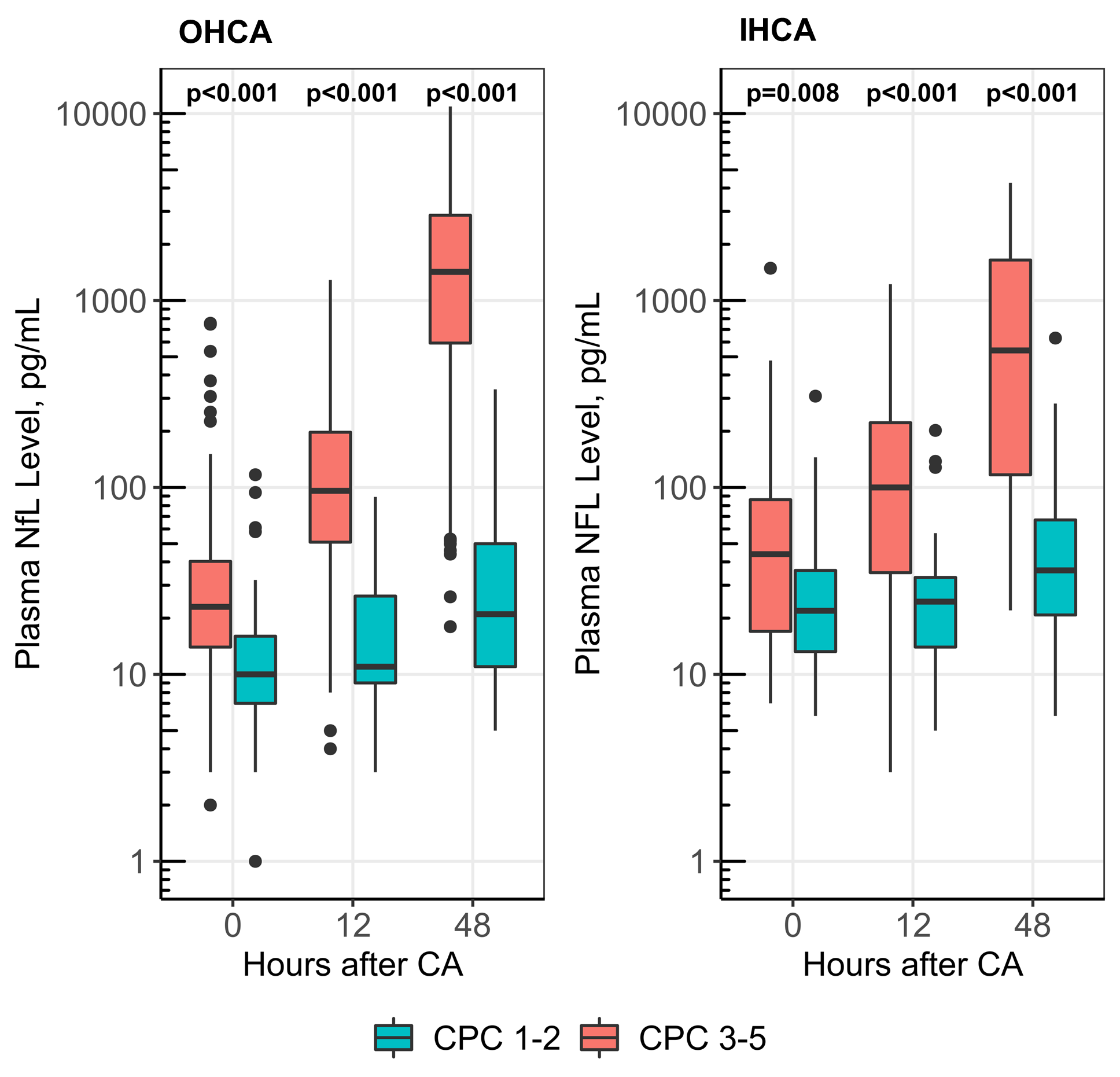


**Fig. S1** Boxplots of neurofilament light (NfL) levels in OHCA and IHCA patients with good (CPC 1-2) and poor (CPC 3-5) outcomes. The boxes show the median and interquartile range (IQR) at the time of ICU admission (0 h), 12 and 48 h after cardiac arrest. The p-values indicate the difference in NfL levels between good and poor outcomes. *OHCA* out-of-hospital cardiac arrest, *IHCA* in-hospital cardiac arrest, *CPC* Cerebral Performance Category


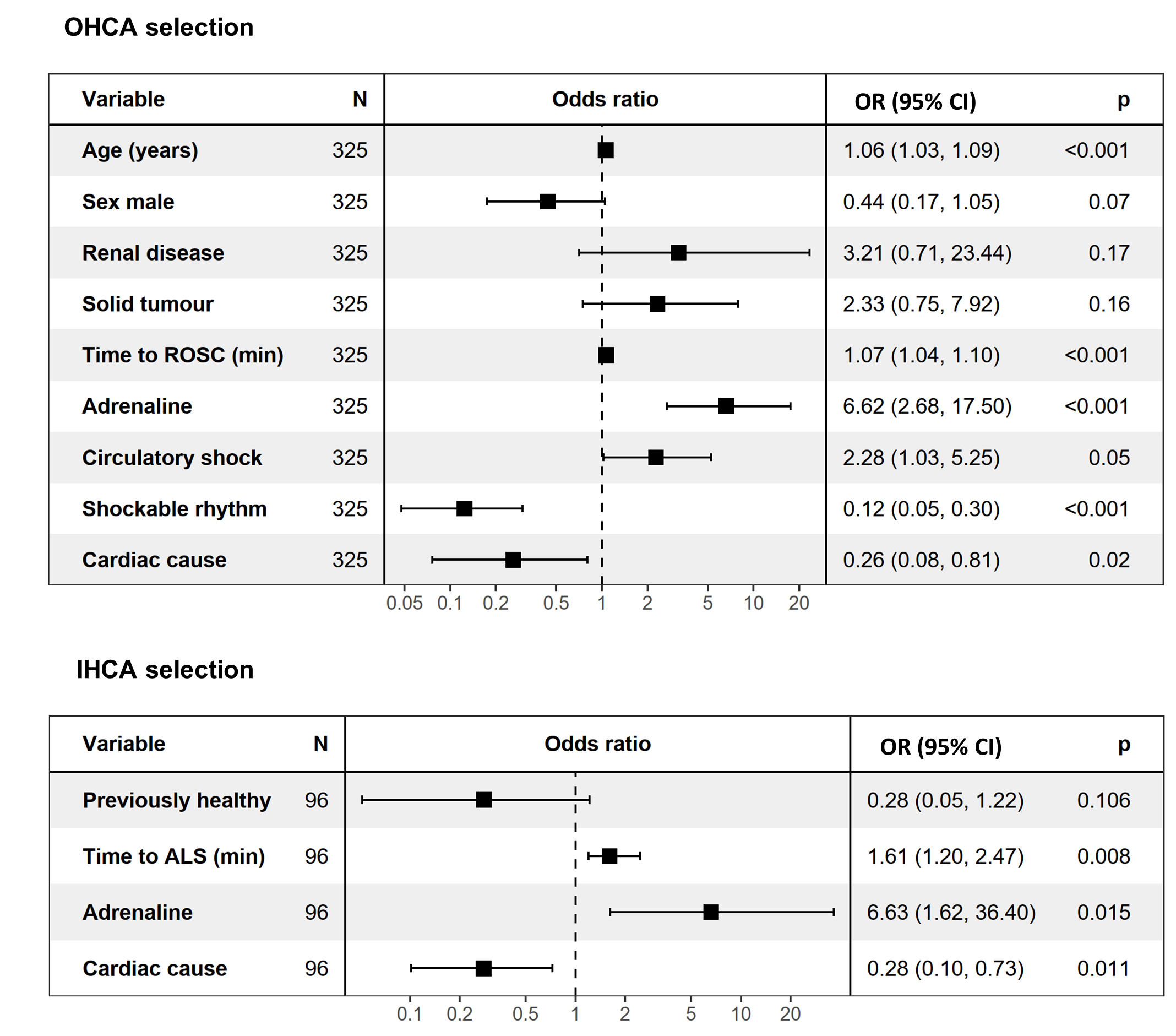


**Fig. S2 A-B** Covariates for prediction of outcome identified separately in the OHCA and IHCA groups with backward stepwise regression. Initially the following variables were included; age, sex, previously healthy, dementia/cognitive impairment, solid tumour, renal disease, diabetes, cerebrovascular disease, myocardial infarction, congestive heart failure, arrhythmia, hypertension, time to ROSC, bystander CPR, witnessed cardiac arrest, time to advanced life support, shockable rhythm, adrenaline, pH, lactate, circulatory shock, and cardiac cause.The backwards elimination was continued for as long as the Akaike information criterion (AIC) decreased. *ROSC* return of spontaneous circulation, *ALS* advanced life support, *OHCA* out-of-hospital cardiac arrest, *IHCA* in-hospital cardiac arrest, *OR* odds ratio.


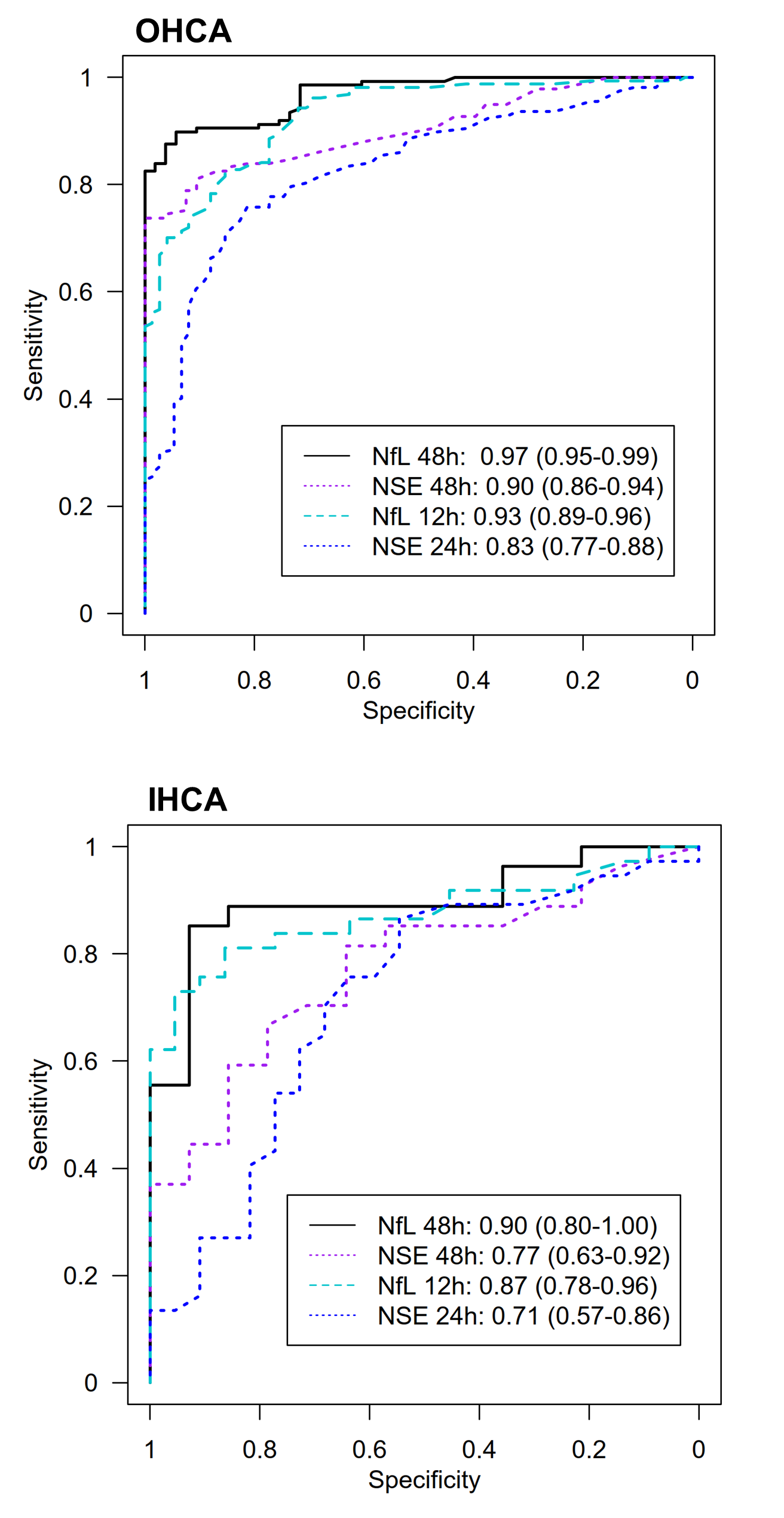


**Fig. S3** Prognostic performance of neurofilament light (NfL) and neuron-specific enolase (NSE) measured with AUROC in a subgroup of OHCA and IHCA patients. As part of the clinical routine samples were collected and analysed for NSE at the local laboratory at 24 and 48h after cardiac arrest. Only patients with both NSE and NfL at 48 h or both NSE at 24 h and NfL at 12 h were included in the analysis. NSE and NfL at 48 h were available in 190 OHCA and 41 IHCA patients. NSE at 24 h and NfL at 12 h were available in 232 OHCA and 59 IHCA patients. *AUROC* area under the receiver operating characteristic curve, *OHCA* out-of-hospital cardiac arrest, *IHCA* in-hospital cardiac arrest
